# Cardiovascular risk in patients with severe mental illness in Italy

**DOI:** 10.1192/j.eurpsy.2020.94

**Published:** 2020-10-26

**Authors:** Virginio Salvi, Andrea Aguglia, Francesco Barone-Adesi, Davide Bianchi, Chiara Donfrancesco, Filippo Dragogna, Luigi Palmieri, Gianluca Serafini, Mario Amore, Claudio Mencacci

**Affiliations:** 1 Department of Neuroscience, ASST Fatebenefratelli Sacco, Milan, Italy; 2 Department of Neuroscience, Rehabilitation, Ophthalmology, Genetics, Maternal and Child Health (DINOGMI), Section of Psychiatry, University of Genoa, Genoa, Italy; 3 IRCCS Ospedale Policlinico San Martino, Genoa, Italy; 4 Department of Translational Medicine, University of Eastern Piedmont, Novara, Italy; 5 Department of Cardiovascular, Endocrine-metabolic Diseases and Aging, National Institute of Health, Rome, Italy

**Keywords:** Bipolar disorder, cardiovascular risk, schizophrenia, severe mental illness

## Abstract

**Background:**

Patients with severe mental illness (SMI), such as schizophrenia or bipolar disorders, are more frequently affected by metabolic syndrome and cardiovascular (CV) diseases than the general population, with a significant reduction in life expectancy. Beyond metabolic syndrome, quantifying the risk of CV morbidity in the long-term may help clinicians to put in place preventive strategies. In this study, we assessed 10-year CV risk in patients with SMI and healthy individuals using an algorithm validated on the Italian general population.

**Methods:**

Patients aged 35–69 years diagnosed with SMI were consecutively recruited from psychiatric acute care units. Single CV risk factors were assessed, and 10-year CV risk calculated by means of the CUORE Project 10-year CV risk algorithm, based on the combination of the following risk factors: age, systolic blood pressure, total and high-density lipoprotein cholesterol, diabetes, smoking habit, and hypertensive treatment. Patients’ data were compared with those from the general population. The 10-year CV risk was log-transformed, and multivariable linear regression was used to estimate mean ratios, adjusting for age, and education.

**Results:**

Three hundred patients and 3,052 controls were included in the analysis. Among men, the 10-year CV risk score was very similar between patients with SMI and the general population (mean ratio [MR]: 1.02; 95%CI 0.77–1.37), whereas a 39% increase in 10-year CV risk was observed in women with SMI compared to the general population (MR: 1.39; 95%CI 1.16–1.66).

**Conclusions:**

In our study, women with SMI were consistently more at risk than the general population counterpart, even at younger age.

## Introduction

Cardiovascular (CV) diseases are the leading cause of death and disability in Western countries. Patients with severe mental illnesses (SMIs) are twice as affected by obesity, diabetes, and metabolic syndrome than the general population and, accordingly, are at similarly increased risk of death due to CV disorders [1].

In the last 10 years, the concept of metabolic syndrome as a risk factor for CV diseases has acquired popularity, and many studies showed elevated rates of metabolic syndrome in patients with SMI compared to the general population [2]. Metabolic syndrome is a pathological entity that identifies a group of people at very high risk for developing CV diseases over time. On the other hand, the use of tools providing an index of risk based on general factors such as age, gender, smoke habits, and lipid levels appears more useful in order to put in place behavioral and nonbehavioral strategies aimed at preventing the occurrence of CV events. For this reason starting from the landmark Framingham study, many others have assessed the 10-year risk of CV diseases in the general populations and in specific subpopulations.

In the last few years, the 10-year CV risk has eventually been assessed in subjects suffering from psychiatric disorders, especially patients with SMI, who are prescribed complex medication regimens, are more prone to inactivity and unhealthy dietary habits independently from socioeconomic status and are likely share genetic factors increasing their vulnerability to metabolic disorders. The majority of reports carried to assess the 10-year CV risk in patients with SMI compared to their reference populations found an increased relative risk of 1.5 in patients with SMI [3–8].

However, results from these studies have limited heuristic value for two main reasons. First, although conducted in Asian, European, and North American countries, they all employed the Framingham risk score algorithm, which was validated on the population of Framingham, USA, several decades ago. In recent years, there has been mounting evidence that the Framingham model overestimates the risk of developing CV disease, especially in high-risk individuals and European populations [9]. Accordingly, risk scores in patients and controls should be calculated employing an algorithm of risk validated on the reference population. Second, patients with SMI usually develop metabolic risk factors well before the general population [10,11]; therefore, a rough estimate of CV risk in a whole sample of patients is of little utility in order to put in place preventive strategies. To this end, risk score calculations in different age groups are needed.

In this study, we aimed to assess the 10-year CV risk in a sample of North Italian patients with SMI stratified by sex and age compared with data from the general population. To do this, we employed a 10-year CV risk algorithm validated on the Italian population.

## Methods

### Study design

The study is a cross-sectional investigation of CV risk factors and estimated 10-year CV risk in patients with SMI compared with data from the Italian general population. The protocol was reviewed and approved by the relevant Ethical Committees (Comitato Etico Milano Area 1, Milan; Comitato Etico Ospedale San Martino, Genoa). To participate, patients had to sign a written informed consent.

### Patients

Patients with SMI consecutively admitted to the psychiatric acute care units of Fatebenefratelli hospital, Milan, and San Martino hospital, Genoa, from May 2017 to April 2018 were recruited.

SMI was defined as having either schizophrenia or bipolar disorder. Patients were evaluated and diagnosed based on the Diagnostic and Statistical Manual of Mental Disorders, 5th edition [12]. For the sake of comparing patients’ data with those from the general population, only those aged 35–69 years were considered for the present study.

Patients were administered a questionnaire to assess sociodemographic and clinical characteristics such as age, gender, education, marital and occupational status, diagnosis, age at onset, number of episodes, presence of lifetime suicide attempts, and current pharmacological treatment. During the index hospitalization, weight, height, and blood pressure were measured. A blood examination assessing fasting glucose, total and high-density lipoprotein (HDL) cholesterol, and triglycerides, was performed. Lifestyles such as current smoking habits and physical exercise were also collected. Physical exercise was classified as absent, mild (<4 h/week), moderate (around 4 h/week), and intense (> 4 h/week).

Educational level was selected as a proxy for socioeconomic position; social class was characterized as primary/middle school (≤8 years; lower education) and high school/university degree (>8 years; higher education).

### Reference population

Data on the reference population (individuals aged 35–69 years and resident in Northern Italy from 2008 to 2012) were obtained from the Osservatorio Epidemiologico Cardiovascolare/Health Examination Survey (OEC/HES). The OEC/HES is part of the CUORE Project, launched by the Italian Ministry of Health and coordinated by the Department of Cardiovascular, Endocrine-metabolic Diseases, and Aging of the Italian National Insitute of health (Istituto Superiore di Sanità- ISS) that provided data for the present analyses.

The CUORE Project monitors CV risk factors, lifestyles, and diseases through different sources of information (population-based registries, longitudinal observational studies, risk assessment by general practitioners, and cross-sectional surveys) to produce a global picture of the population’s health and to identify priority areas for treatment and preventive actions [13].

The 10-year CV risk score from the general population of Northern Italian regions, based on risk factors collected within the OEC/HES 2008–2012 survey, was considered stratified by gender, age, and educational level groups.

### 10-year CV risk assessment

To assess CV risk, we employed the same algorithm developed within the CUORE Project [14,15] and applied to OEC/HES participants [13]. The CUORE Project 10-year CV risk algorithm is based on the combination of seven risk factors (age, systolic blood pressure, total and HDL cholesterol, diabetes, smoking habit, and hypertensive treatment) and predicts 10-year fatal, and nonfatal coronary and cerebrovascular incidence [14,15]. To assess the relation of risk factors, considered together, to the 10-year CV disease risk, multivariate analyses were performed. Starting from a basic Cox model that included age, systolic blood pressure, and dichotomic smoke habit, the other CV risk factor variables, when statistically significant in the univariate analyses and not presenting multicollinearity with the other variables in the model under study, were progressively included. The 10-year CV disease individual risk score models were implemented including continuous values of risk factors when available. All multivariate models were evaluated for accuracy by assessing the area under the receiver operating characteristic curve and performing Hosmer-Lemeshow test [15].

### Statistical analyses

Means and standard deviations were used to describe continuous variables, and counts and percentages were used for categorical variables. The mean levels of CV risk factors were sex- and age-standardized using the age structure of the general population, aged 35–69 years, resident in the Northern Italian regions in 2010. As a preliminary analysis showed that the difference of CV risk score between the two groups was constant among the different age groups on the multiplicative but not on the additive scale, CV risk score was log-transformed, and multivariable linear regression was used to estimate mean ratios, adjusting for age, and education.

Analyses were performed using Stata 15 Software (Stata Corp, College Station, TX).

## Results

A total of 300 patients and 3,052 controls from the general population of Northern Italy were included in the study.

Mean age was comparable between patients and controls in both sexes. Gender was evenly distributed in patients and controls. Sociodemographic and clinical variables are shown in Table 1. Looking at CV risk factors, both men and women with SMI had higher triglycerides, lower HDL cholesterol levels, and smoked more cigarettes/day than controls. On the other hand, men and women from the general population had higher total cholesterol levels than patients. Males from control group also showed higher systolic blood pressure than female patients. CV risk factors in patients and controls are shown in Table 2.

**Table 1.** Sociodemographic and clinical characteristics of patients with severe mental illness.

Females, *n* (%)	150 (50.0)
Age (years), mean ± SD	51.9 ± 10.7
Education (years), mean ± SD	11.3 ± 4.0
Marital status	
Single	175 (58.3)
Married	69 (23)
Divorced	44 (14.7)
Widowed	12 (4.0)
Occupational status	
Unemployed	148 (49.3)
Blue collar	23 (7.7)
White collar	60 (20.0)
Housewife	6 (2)
Student	3 (1)
Retired	60 (20)
Diagnosis	
Bipolar disorder I	115 (38.3)
Bipolar disorder II	61 (20.3)
Schizophrenia	124 (41.3)
Duration of illness (years), mean ± SD	23.1 ± 13.0
At least one suicide attempt, *n* (%)	96 (32.0)
Pharmacological treatment, *n* (%)	
*Antipsychotics*	269 (89.7)
*Mood stabilizers*	177 (59)
*Antidepressants*	68 (22.7)

Abbreviation: SD, standard deviation.

**Table 2.** Cardiovascular risk factors in patients and controls.

	Males		Females	
	Patients	Controls	Patients	Controls
	*N* = 150	*N* = 1528	*N* = 150	*N* = 1524
Age (years), mean ± SD	49.9 ± 4.60	50.5 ± 3.1	50.7 ± 4.7	51.0 ± 2.9
Systolic blood pressure (mmHg), mean ± SD	123.7 ± 10.5	132.7 ± 15.9	122.6 ± 14.6	125.9 ± 16.0
Diastolic blood pressure (mmHg), mean ± SD	77.8 ± 7.2	86.2 ± 9.6	75.5 ± 8.2	80.0 ± 9.2
Total cholesterol (mg/dl), mean ± SD	174.1 ± 31.5	213.3 ± 41.6	193.9 ± 41.2	215.2 ± 40.6
HDL cholesterol (mg/dl), mean ± SD	43.6 ± 12.2	52.4 ± 12.1	52.3 ± 13.6	64.4 ± 15.1
Triglycerides (mg/dl), mean ± SD	141.3 ± 82.1	130.7 ± 83.2	118.0 ± 61.6	99.0 ± 49.9
Glucose (mg/dl), mean ± SD	95.8 ± 21.3	97.5 ± 16.9	90.1 ± 25.4	89.7 ± 17.6
Body mass index, mean ± SD	27.6 ± 4.3	27.0 ± 4.2	25.7 ± 6.4	25.7 ± 5.0
Current smokers (%)	64.7	25.9	51.3	20.4
Tobacco cigarettes/day (*n*)*, mean ± SD	22.8 ± 11.6	16.1 ± 9.2	18.6 ± 9.2	10.8 ± 6.1
10-year CV risk (%)	6.3 ± 3.7	6.5 ± 4.2	3.1 ± 2.9	2.2 ± 1.9

All variables were age-standardized using the age structure of the general population * in smokers.

Abbreviations: CV, cardiovascular; sd, standard deviation.

**Figure 1.**
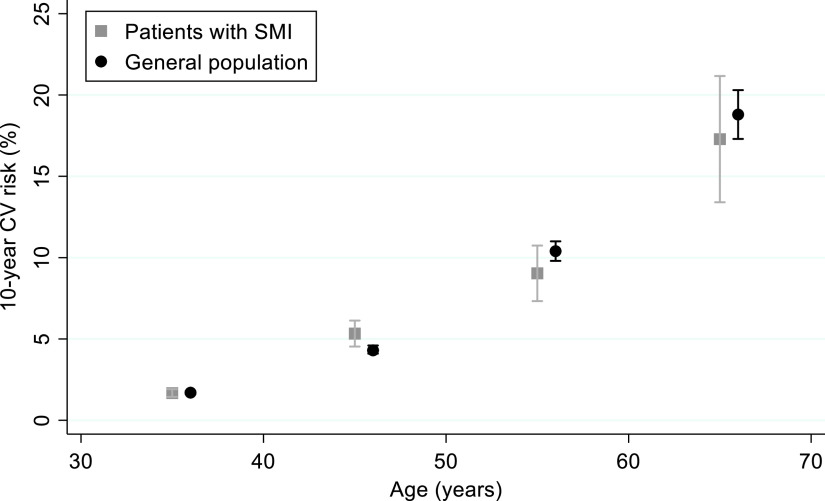
Ten-year cardiovascular risk in patients with severe mental illness compared to the general population. Men.

**Figure 2.**
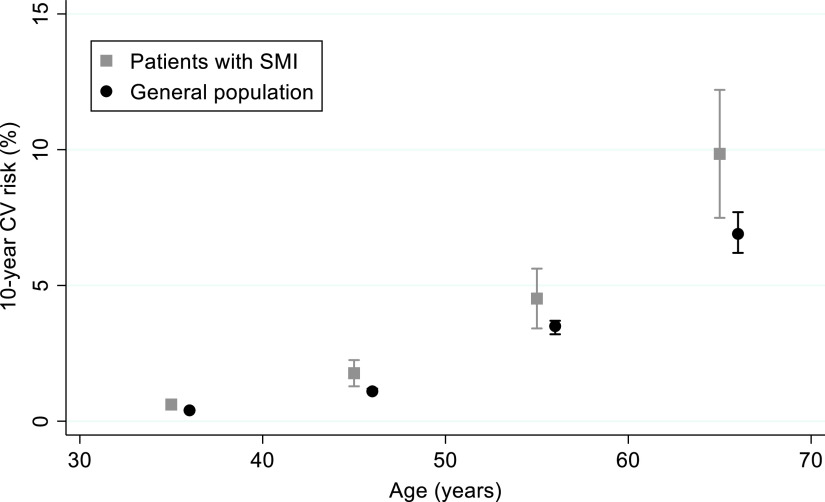
Ten-year cardiovascular risk in patients with severe mental illness compared to the general population. Women.

Among men, the 10-year CV risk score was very similar between patients with SMI and the general population (mean ratio [MR]: 1.02; 95%CI 0.77–1.37) (Figure 1 and Table 3). On the opposite, compared to the general population, women with SMI had a 39% increase in 10-year CV risk (MR: 1.39; 95%CI 1.16–1.66). Such an increase was evident in all age classes (Figure 2). These findings did not change after adjusting for participants’ educational level in both sexes (Table 3).

**Table 3.** Adjusted 10-year cardiovascular risk in patients with severe mental illness and controls.

		Model 1^a^	Model 2^b^
		Mean ratio (95%CI)	Mean ratio (95%CI)
Men	General population	1 (ref.)	1 (ref.)
	Patients	1.02 (0.77–1.37)	1.02 (0.90–1.16)
Women	General population	1 (ref.)	1 (ref.)
	Patients	1.39 (1.16–1.66)	1.41 (1.28–1.56)

a Adjusted by age.

b Adjusted by age and education.

## Discussion

In this study, we assessed 10-year CV risk in patients with SMI and compared it with a large sample from the general population dwelling in the same regions. To this end, we grouped patients and controls according to sex and age, which are the main drivers of CV risk, being CV morbidity higher in the elderly and males [16].

As expected, the analyses showed that the estimated CV risk increases with age in both sexes. Educational level is a proxy for socioeconomic status, a sociodemographic factor known to influence CV risk; plus, well-educated people are more informed and more frequently follow guidelines and medical advice. The previously reported OEC/HES study conducted in Italy reported a small but significant reduction in CV risk factors in patients with a higher educational level [13]. Therefore, we adopted the same cutoffs and controlled our results for educational level, which, however, did not affect the results: the estimated CV risk was comparable in SMI patients with low or high educational attainment.

The vast majority of published studies have assessed the 10-year CV risk through the Framingham risk algorithm [4–8,17–19]. However, in a prospective study aimed at validating several risk algorithms on the UK population, the use of the Framingham algorithm overestimated CV risk in subjects with SMI, especially in males [20]. The inaccurate risk prediction may reflect differences between historical North American people and contemporary individuals from other countries; since by the time the Framingham model was developed, CV risk factors such as hypercholesterolemia or hypertension were not routinely treated, eventually leading to a higher number of CV events over time [9]. For these reasons, the authors recommended the preferable use of CV risk algorithms validated on the local general population. In keeping with this suggestion, we employed the CUORE Project algorithm [14,15] that was applied also to OEC/HES participants [13].

The main finding of our study is the 40% increase in estimated CV risk in women with SMI compared with women from the general population. In fact, in our sample, women with SMI were consistently more at risk than their general population counterpart, even at younger ages. Similar results were found in the CATIE study, where the mean 10-year risk of coronary heart disease morbidity was 9.4% in males and 6.3% in females, yet a 50% increase of relative risk was found in females with schizophrenia compared to 34% in males [5]. The results are also in line with the findings from a recent Scottish retrospective cohort study in which, aside from a generally higher incidence in males, the relative risk of ischemic heart disease and stroke associated with schizophrenia, bipolar disorders, or major depressive disorders was larger in females [21].

Patients with SMI frequently adopt at-risk lifestyles, such as unhealthy eating, low physical activity, or absence thereof [22,23]. Moreover, the use of psychotropic medications, especially second-generation antipsychotics but also some mood stabilizers and anti-histaminergic antidepressants, further increases the incidence of obesity and other metabolic issues, leading to CV morbidity and mortality [24–26]. Lastly, due to their reduced access to medical care, key CV risk factors remain underscreened and undertreated in this population. The importance of a proper CV screening was made clear by Crump et al. study, in which the association between bipolar disorder and CV mortality was weaker in patients with a prior CV diagnosis, whereas in those without a prior CV diagnosis, the risk CV death was higher [27].

Beyond general risk factors, there is evidence that gender-related specific factors play a role. Exogenous hormone use such as contraceptive pills showed to increase the risk of myocardial infarction and stroke [28], and hormone replacement therapy in postmenopausal women increases stroke risk by about one-third [29]. Finally, women are less likely to receive guidance regarding preventive treatments, such as healthy behavior changes, lipid-lowering therapy, and aspirin use [30].

In our sample, the effect of SMI on estimated CV risk was higher among women, independently from educational level. Looking at the risk profile, females with SMI displayed higher triglycerides and lower HDL cholesterol levels than healthy individuals. They also smoked more cigarettes per day. On the contrary, other factors such as total cholesterol, glucose, and blood pressure were comparable in women with or without SMI. The concordance between these results and findings from the literature is extremely high, with matching results in European, North American, and Asian populations [4,6–8,31,32], suggesting that these parameters are not primarily affected by factors related to mental illness.

Examining the risk profile in men with SMI, we found, akin to women, higher triglycerides, lower HDL cholesterol, and more cigarette smoking compared with the control group. However, the increased risk in males is likely offset by lower systolic blood pressure in patients with SMI. Several studies found lower blood pressure values in patients with schizophrenia and bipolar disorders compared with general population samples [4,31,32]. In the most recent Danish study, the authors argued that the finding was surprising, since subjects in their healthy comparison group were blood donors and likely with a healthy lifestyle [32]. A tentative explanation may reside in the use of hypotensive medications: either first- or second-generation antipsychotics, particularly chlorpromazine, clozapine, or quetiapine, are associated with reduced blood pressure and orthostatic hypotension [33].

This is a multicenter study investigating CV risk on a relatively large sample of patients with SMI. Since patients were consecutively recruited, selection bias seems unlikely. General population samples used as control group were drawn from the same Northern Italian regions of the patients, thus increasing the reliability and robustness of the findings. Furthermore, as noted above, the use of an algorithm already validated in the Italian general population likely improves the accuracy of this prediction. However, some potential limitations should be acknowledged. First, this study did not prospectively follow patients to assess incident CV outcomes; therefore, it simply provides an estimate of the CV risk, not assessing the actual risk. Second, likewise the Framingham CV risk tool, the CUORE project CV risk algorithm was validated on the general population without SMI; therefore, it may not accurately catch the effect of SMI-specific exposures on CV risk; it would be appropriate for general population risk assessment tools to incorporate specific mental health measures. Having selected only subjects between 35 and 69 years of age, we cannot infer on CV risk in young or old patients; therefore, the results may not be generalized to all patients with SMI. Finally, the cross-sectional design of the study does not allow inferences on the causal relationship between the variables; therefore, prospective studies are needed to tease out the relative contribution of lifestyle and psychotropic medications on CV risk.

Our finding of an increased estimated CV risk in women with SMI is of utmost relevance. CV disease accounts for one-third of all deaths among females worldwide [34]. CV morbidity has been on the rise especially in vulnerable populations, such as socially disadvantaged and marginalized women with low income, increased stress, or childhood adversities [30].

CV risk is the main driver of the observed reduction in life expectancy in people with SMI [1]. Preventive strategies should include frequent monitoring, lifestyle interventions such as encouraging healthy eating and physical activity [35], and modifying psychopharmacological treatment by switching to medications with negligible effects on weight and metabolism. Being these strategies not easily accomplished by SMI patients, some ways to overcome difficulties may be the enforcement of mental health services with integrated care managers or allocating funds to implement lifestyle modification programs in community mental health centers.

## Data Availability

The data that support the findings of this study will be made available upon request to the authors.
